# Effect of Oil Palm Leaf Meal (*Elaeis guineensis*) on Growth Performance, Haematology and Carcass Characteristics of West African Dwarf Sheep

**DOI:** 10.1002/vms3.70581

**Published:** 2025-08-27

**Authors:** Paulinus Ogbonnaya Essen, Godwin Nnamdi Njoku, Godswill Uzoma Onya, Blessing Ngozi Zachary, Dondo Stephen Donkoh, Timothy Tartenger Kuka, Benjamin Adjei‐Mensah

**Affiliations:** ^1^ Regional Centre of Excellence in Avian Science University of Lome Lome Togo; ^2^ Department of Animal Science University of Ghana Legon Ghana

**Keywords:** carcass, growth performance, haematology, oil palm leaf meal, WAD Sheep

## Abstract

**Background:**

Nigerian ruminant farmers face poor animal nutrition during dry seasons, affecting productivity.

**Objectives:**

A 90‐day study evaluated the effect of oil palm leaf meal (OPLM) supplementation on the performance of West African Dwarf (WAD) weaner sheep.

**Methods:**

Fifteen WAD sheep aged 4–5 months were randomly assigned to three dietary treatments in a completely randomised design. Diets were supplemented with 0%, 10% and 20% inclusion levels of OPLM. At the end of the study, feed intake, weight gain, feed conversion ratio (FCR), haematology and carcass characteristics were determined. Two millilitres of blood samples were collected from three sheep per treatment with a syringe and needle from the jugular vein into bottles containing ethylenediaminetetraacetic acid (EDTA) for haematology analysis. Three sheep per treatment were randomly selected, starved of feed for 12 h, slaughtered and the carcass was cut into parts and weighed using a sensitive scale. The weights of internal organs were expressed as a percentage of the live weight.

**Results:**

The results showed that feed intake was significantly higher in the 20% OPLM diet and lower in the 10% OPLM diet and had the best FCR and average daily body weight gain of 11.36 and 80.37 g, respectively. Significant differences (*p* < 0.05) were observed among the groups on haematology and carcass characteristics.

**Conclusion:**

Therefore, OPLM can be included in the sheep diet up to 10% without compromising performance.

## Graphical abstract

Oil palm leaf meal (OPLM) is a promising feed ingredient for sheep, enhancing nutrient absorption, rumen health, and growth. A 10% inclusion improved weight gain and feed efficiency, while 20% had negative effects on weight and carcass traits. OPLM supplementation positively influenced blood parameters and meat production efficiency, making it a viable option for sustainable sheep nutrition.

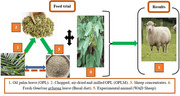

## 1 Introduction

Sheep are multi‐functional animals that play a significant role in the economy and nutrition of both rural and urban dwellers (Oluwatomi 2013). Domesticated sheep are now found throughout the continents, including Nigeria, which has a population of 234.5 million (FAO 2005; NP 2024). In Nigeria, sheep rearing is primarily traditional, characterised by extensive or semi‐intensive management systems where animals rely on natural grazing, crop residues, and household scraps, often with limited supplementation. This system lacks structured nutritional planning, leading to inadequate feeding practices that may result in nutrient deficiencies, slow growth, and lower productivity. Consequently, there is a growing need to explore non‐conventional feedstuffs that are nutritious, cost‐effective, and not in high competition with human food sources (Ahamefule et al. 2005).

Various unconventional feed resources have been tested on ruminant species, including cattle, sheep and goats. These alternative ingredients include soursop (Kuka and Anayo 2017), almond leaves and hulls (Elahi et al. 2017), agro‐industrial by‐products (Correddu et al. 2020), water hyacinth (Azeez et al. 2021; Yared et al. 2024), insect meals (Renna et al. 2023), macro‐ and microalgae (Harahap et al. 2024), sorghum bicolor and bitter vetch (Boukrouh et al. 2024a, 2024b; Petrova et al. 2025). Studies have consistently demonstrated that these unconventional feedstuffs are readily available, locally sourced, and help reduce competition between livestock and humans for conventional feed ingredients. Additionally, they have shown no adverse effects on farm animals. Instead, they enhance performance, lower production costs and improve overall health and welfare. Their integration into livestock nutrition represents a sustainable approach to feed management, contributing to environmental conservation and resource efficiency. Based on these findings, oil palm leaf meal (OPLM) is expected to exhibit similar benefits, making it a viable alternative feed ingredient for ruminants.

One of the problems facing ruminant farmers in Nigeria is poor nutrition, especially during the dry season, when there is a decline in the supply and quality of feed for the animals. The tropics are characterised by seasonal shortages in the quality and quantity of forage from natural pastures, which have continuously led to low productivity in the ruminant industry, particularly for peasant farmers who cannot afford the cost of forage conservation (Ukanwoke et al. 2013). To enhance production, sheep fed on forages are supplemented with concentrates, which have, in turn, inflated production costs. Therefore, using cheaper, readily available, easily accessible and unconventional feedstuffs such as oil palm leaf meal may help reduce production costs while improving animal production and performance.

Ammoniation of oil palm frond (OPF) with non‐protein nitrogen (N) sources has been shown to improve the nutritional value and digestibility of OPF in ruminants. Mookiah et al. (2022) evaluated the effect of treating OPF without (control) or with different urea levels (1%–5%) on chemical composition and in vitro gas production, digestibility and fermentation properties using goat rumen fluids. The results showed that ammoniation of OPF with urea improved its nutritional value and in vitro rumen fermentation profiles in goats. The impact was more pronounced for 4% or 5% urea‐treated OPF. Pin et al. (2017) fed fungal (*Lentinussajor‐caju*) treated oil palm frond in total mixed ration and reported no significant effect on performance, carcass traits, meat quality and muscle chemical composition on finishing crossbred male goats (Thai Native×Anglo Nubian).

When oil palm compounds were used to replace para‐grass in female crossbred goats for 28 days, the authors observed increased dry matter intake, plasma cholesterol level, body weight gain and increased microbial number and activity suitable for acetate production. Their findings suggested that oil palm by‐products are partly useful as a replacement for para‐grass and are a useful energy source for small ruminants (Buranakarl et al. 2020). In the study by Nuryawan and Risnasari (2023), it was confirmed that oil palm leaves (OPL) have traditionally been used primarily as a feed source for ruminants, particularly cattle. However, recent research highlights their potential applicability beyond cattle, suggesting that OPL could be incorporated into the diets of other ruminants, such as sheep and goats. This expansion in usage is driven by ongoing studies on its nutritional profile, digestibility, and impact on animal health and productivity.

The oil palm tree has a distinctive single, unbranched trunk that can reach up to 20 m in height. The leaves are feather‐like and can grow between 9 and 16 feet long. These leaves form a crown at the top of the trunk, giving the tree its characteristic look. Oil palm leaves are a widely available farm waste in Nigeria. They have been identified as a potential feed for ruminants and other herbivorous animals (FAO 2005). Oil palm leaves contain a variety of phytochemicals, including flavonoids, phenolic acids, alkaloids, saponins, tannins and vitamin E isoforms. These phytochemicals possess antioxidant properties that contribute to potential health benefits, including anti‐inflammatory, anti‐diabetic and cardiovascular protective effects (Egbunike and Nworgu 2013; Phin et al. 2013). The abundance of this plant around homesteads and villages, combined with reasonable levels of certain valuable nutrients, appears not to have been fully utilised or exploited in ruminant feeding.

It is hypothesised that OPLM can enhance blood profile, improve feed conversion ratio (FCR), support growth performance and increase slaughter yield. This hypothesis is based on the presence of antinutritional factors such as tannins, which have been reported to influence digestibility and overall performance of small ruminants (Akinyeye et al. 2011; Al Rharad et al. 2025). Therefore, the current study was designed to evaluate the nutritive value of oil palm leaves and assess their effects on the growth performance, haematological parameters and carcass characteristics of West African Dwarf sheep.

## 2 Materials and Methods

### 2.1 Experimental Site

The research was conducted at the Sheep and Goat unit of the Teaching and Research Farm, Federal College of Agriculture Ishiagu, Ivo Local Government Area, Ebonyi State, Nigeria.

### 2.2 Sourcing and Processing of Test Ingredient

The oil palm leaves were harvested from the college's oil palm plantation and air‐dried on a concrete floor for 7 days under shade (air‐drying), ensuring gradual and consistent moisture reduction. The drying period was monitored, and the leaves were stirred each day to maintain uniform dehydration. After drying, the leaves were ground using a hammer mill and bagged for sheep feed formulation.

### 2.3 Experimental Animals and Management

A total of 15 West African Dwarf Weaner Sheep, aged 4–5 months and weighing an average of 8.53 ± 1.4 kg, were selected from the college flock for the study. Before the trial, the animals were dewormed using ivermectin and administered antibiotics to prevent secondary bacterial infections that could arise from environmental stress. This measure was taken to ensure the experimental animals were in optimal health before the study commenced, minimising variability in results due to health complications and ensuring reliable data collection. Each animal was housed individually in a well‐ventilated concrete floor pen equipped with feeding and watering troughs. The floor of the experimental site was washed with detergent and water and disinfected before the animals were introduced into the pen. Adequate management practices were implemented to prevent any outbreak of disease during the duration of the project.

### 2.4 Data Collection

After a 1‐week holding period for acclimatisation to experimental conditions, the 15 WAD Weaner sheep were randomly divided into three groups, each consisting of five animals. The groups were randomly assigned to three experimental diets in a completely randomised design (CRD). The ingredient and nutrient composition of the diet fed to the animals is shown in Table 1. The dietary supplementation levels of OPLM comprise 0%, 10% and 20%. The experimental diets were formulated to meet the nutrient requirements of weaned sheep. The experiment lasted 90 days. Potable drinking water and feed were offered ad libitum daily, and leftovers were recorded the next day by weighing the remnants to determine the voluntary daily feed intake. Fresh Gmelina leaves were offered later in the afternoon as forage. Initial weights of the animals were recorded at the beginning of the trial and weekly thereafter.

**TABLE 1:** Gross composition of the experimental diet for WAD sheep and proximate composition of oil palm leaf meal (OPLM).

Ingredients	Composition (%)
Cassava peel meal	45.0
Maize offal	10.0
Palm kernel cake	14.0
Brewer's dried grain	25.0
Molasses	2.0
Bone meal	3.0
Table salt	1.0
Total	100
Calculated nutrients	
Metabolizable energy (kcal/kg)	2528.70
Total digestible nutrient (%)	32.30
Crude protein (%)	12.92
Crude fibre (%)	12.0

Proximate composition of oil palm leaf meal (OPLM) and experimental diet		
Parameters	OPLM	Experimental diet
Dry matter	89.80	90.25
Moisture content	5.75	9.75
Crude protein	9.92	13.48
Crude fibre	22.89	29.09
Ash	12.53	15.05
Ether extract	3.90	0.64
Nitrogen‐free extract	43.61	43.65
Metabolizable energy (kcal/kg)	2705.50	1965.20

The proximate composition of OPLM and experimental diet, based on a dry matter basis, revealed a high content of dry matter, nitrogen‐free extract, crude fibre, and ash, while exhibiting a low crude protein and low ether extract. The calculated metabolizable energy value was 2705.50 and 1965.20 kcal/kg, respectively, indicating its potential as a viable feed ingredient/diet (Table 1).

At the end of the experimental period, 2 mL of blood samples were collected from three well‐restrained sheep (ewe) per treatment to determine haematological parameters. Blood was collected using a sterile disposable syringe from the jugular vein, into an anticoagulant tube. Blood samples in ethylenediaminetetraacetic acid (EDTA) bottles and the oil palm leaf meal were taken to the veterinary laboratory at Michael Okpara University of Agriculture, Umudike, for haematological and proximate analysis. The proximate composition of the test ingredients was carried out according to the method of AOAC (2000).

A total of nine sheep were randomly selected from the flock, specifically three per treatment, at the end of the experiment for carcass and internal organ evaluation. The selected animals were kept separate from the rest of the flock and were provided only with water ad libitum for 12 h before slaughter. On the day of evaluation, they were taken to the institution's mini‐abattoir, where they were weighed to determine live weight (LW) before slaughter. After slaughter, the experimental animals were scalded, and their internal organs were removed, and the carcass weight (CW) was recorded. The head, legs, kidneys, heart and liver were left on the carcass and included in the CW. Dressing percentage was calculated as the ratio of the carcass weight to the live weight. All internal organs (heart, lungs, kidneys, liver, spleen, intestines) and other parts (head, neck, thighs, legs) were weighed and expressed to dressed weight.

### 2.5 Statistical Analysis

All data collected for each parameter were subjected to analysis of variance (ANOVA) appropriate for CRD using the general linear model of SPSS (2004) (SAS, 2010). Prior to conducting ANOVA, the assumptions of normality and homogeneity of variances were assessed. Normality of residuals was evaluated using the Shapiro–Wilk test, and homogeneity of variances was tested using Levene's test. Where assumptions were met, treatment means were compared using Duncan's multiple range test (DMRT) at a significance level of *p* < 0.05.

## 3 Results and Discussion

### 3.1 Results

The growth performance results of West African Dwarf Sheep (ewe) fed OPLM are presented in Table 2. Significant differences (*p* < 0.05) were observed across all treatment groups, except for total feed intake and average daily feed intake. Final body weight and weight gain were the highest with a corresponding low FCR in the 10% group and lowest in the 20% group, with a higher FCR.

**TABLE 2:** Growth performance of WAD sheep fed diets containing varying levels of oil palm (*Elaeis guineensis*) leaf meal (OPLM).

Parameters	(0% OPLM)	(10% OPLM)	(20% OPLM)	SEM	*p* value
Initial body weight (kg)	8.533	8.520	8.533	0.004	0.081
Final body weight (kg)	12.767b	13.020a	12.300c	0.211	<0.00001
Total weight gain (kg)	4.233b	4.500a	3.767c	0.214	<0.00001
Daily weight gain (kg)	0.076b	0.080a	0.067c	0.004	0.004
Total feed intake (kg)	53.700	50.000	54.067	1.30	0.617
ADFI (kg)	0.959	0.893	0.966	0.023	0.846
Feed conversion ratio	12.69b	11.36c	14.39a	0.88	0.036

*Note*: Means within the row having different lowercase letters differ significantly from one another (*p* < 0.05).

Abbreviations: ADFI, average daily feed intake; SEM, standard error of mean.

The haematological indices of West African Dwarf Sheep fed OPLM are shown in Table 3. Significant differences (*p* < 0.05) were noted among the treatment groups for all observed haematological indices. Pack cell volume (PCV), red blood cell (RBC), haemoglobin (Hb) and white blood cell (WBC) values were higher in the treatment groups compared to the control group, while platelets, mean corpuscular haemoglobin (MCH), mean corpuscular volume (MCV) and mean corpuscular haemoglobin concentration (MCHC) values were higher in the control group than in the supplemented groups.

**TABLE 3:** Haematological parameters of WAD sheep fed oil palm (*Elaeis guineensis*) leaf meal (OPLM).

Parameters	(0% OPLM)	(10% OPLM)	(20% OPLM)	SEM	*p* value
PCV (%)	29.4b	32.25ab	33.30a	0.59	0.0001
Hb (g/dL)	8.35b	9.4ab	9.60a	0.21	0.0001
RBC (X10^6^/mcL)	2.08b	2.53ab	2.60a	0.09	0.0008
MCHC (g/dL)	300.50a	299.25ab	286.65b	2.22	0.0001
MCH (pg)	40.35a	39.50b	40.26ab	0.15	0.0004
MCV (fL)	134.80a	132.7b	132.85ab	0.35	0.0003
WBC (X10^3^/mcL)	184.0c	193.82b	198.55a	2.15	0.0003
PLT (X10^3^/mcL)	13.23a	12.60ab	9.08b	0.65	0.0002

*Note*: Means within the row with different lowercase letters differed significantly from one another (*p* < 0.05).

Abbreviations: Hb, haemoglobin; MCH, mean corpuscular haemoglobin; MCHC, mean corpuscular haemoglobin concentration; MCV, mean corpuscular volume; PCV, pack cell volume; PLT, platelets; RBC, red blood cell; SEM, standard error of mean; WBC, white blood cell.

The results of the carcass and internal organ characteristics of West African Dwarf Sheep fed varying levels of OPLM are presented in Table 4. All parameters showed significant differences (*p* < 0.05), indicating that the experimental animals were influenced by the test ingredients. The 10% group had a higher value for the carcass.

**TABLE 4:** Carcass and internal organ characteristics of WAD sheep‐fed diets containing varying levels of oil palm (*Elaeis guineensis*) leaf meal (OPLM).

Parameters	0% OPLM	10% OPLM	20% OPLM	SEM	*p* values
Live weight (kg)	12.767ab	13.333a	12.100b	0.356	0.026
Carcass weight (kg)	8.407c	10.763a	9.307b	0.687	0.0003
Dressed percentage (%)	56.05b	62.66a	58.58ab	2.69	0.039
Primal cut‐parts					
Thighs (%)	18.35b	20.34a	19.91a	0.53	0.018
Neck (%)	4.00b	4.97a	3.71b	0.15	0.0001
Legs (%)	3.27c	3.84a	3.61b	0.09	0.005
Offal/organs					
Head (%)	7.29c	9.05a	8.40b	0.24	<0.0001
Lungs (%)	1.26b	1.48a	1.32b	0.06	0.028
Spleen (%)	0.15b	0.17ab	0.19a	0.01	0.0039
Kidneys (%)	0.38b	0.50a	0.40ab	0.01	<0.0001
Liver (%)	2.00b	2.49a	2.35a	0.08	0.0028
Intestine (%)	30.92b	34.55a	30.83b	0.99	0.005
Heart (%)	0.55c	0.64a	0.59b	0.02	0.017

*Note*: Means within the row with different lowercase letters differed significantly from one another (*p* < 0.05).

Abbreviations: OPLM, oil palm leaf meal; SEM, Standard error of mean.

### 3.2 Discussion

The result showed high dry matter and low moisture content, which indicates good nutritive value of the test ingredient and a prolonged shelf life in terms of storage duration (Akinsola et al. 2021; Umeoka 2024); appreciable crude protein amount makes it a good feed ingredient since protein is the most expensive nutrient in animal feeds (Zirari et al. 2024). High crude fibre makes it a good feed ingredient for ruminants as it helps maintain the rumen's health and promotes digestive efficiency and nutrient absorption (Karoly 2011). High ash indicates the availability of minerals that are essential for production performance (Marzuki et al. 2018; Umeoka 2024). Low ether extract points towards the suitability and positive quality of the test ingredient (Kuka and Anayo 2017; Romes et al. 2019). Nitrogen‐free extract shows the energy required by the animals to carry out metabolic and daily activities (Zirari et al. 2024). Based on this result, OPLM is a good feed ingredient for ruminants, especially sheep.

Furthermore, the values obtained in this study on dry matter, moisture content, ether extract, crude protein and nitrogen‐free extract are closely related to maize, which is a popular feed ingredient for all animals. These values are also similar to those reported by Kuka et al. (2017) who used *Annona muricata* leaf meal in feeding goats and the values included 93.91%, 6.09%, 4.31%, 20.41% and 42.39%, respectively, but lower values on ash (10.62%). Ikyume et al. (2018), on the other hand, reported feeding four different leaves (*Gliricidia sepium*, *Leucaena leucocephala*, *Centrosema pubescence* and *Moringa oleifera*) and obtained 87%­–88% dry matter, 4%­–8% ether extract, 9%­–12% crude fibre, 8%­–11% ash and 20 – 21% crude protein. All the values fell within the values recorded in this study, except for crude protein and crude fibre, which appeared higher and lower, respectively, which could be attributed to the class of the leaves.

There were significant differences in the final body weight of the sheep, with the highest and lowest values observed in 10% OPLM and 20% OPLM, respectively. These differences are likely due to the treatment effect, given that the diets were isoenergetic and isoproteinic. The only variation observed is related to the inclusion level of OPLM. According to Adams (2019), feed intake is the single most important factor in determining the growth rate of animals. However, this study differed from that view as the feed intake was lower in the 10% group than in the 20% group. Total feed intake and average daily feed intake were the lowest in 10% OPLM and higher in 20% OPLM, respectively. Feed intake has been observed to be governed by many factors like dietary crude protein, palatability, smell, gut fill, taste, health, breed/species and other environmental conditions (Ukanwoke, Ibeabuchi, and Ukachukwu 2009). There were significant changes in body weight gain and FCR, with the highest and best values observed in 10% OPLM and the lowest and poorest values in 20% OPLM. The total feed intake and FCR in the 10% OPLM group indicate that the animals efficiently converted feed into body mass. These findings suggest that OPLM inclusion at 10% improves sheep growth performance, whereas higher inclusion levels (20%) negatively impact weight and carcass traits.

Sheep generally have lower efficiency in valorising alternative feed resources compared to other ruminants, such as goats. For instance, Boukrouh et al. (2024b) reported that goats receiving up to 30% bitter vetch showed no significant impact on growth performance, while 20% OPLM inclusion in sheep diets resulted in reduced body weight and carcass parameters. This highlights species‐specific differences in nutrient utilisation and adaptability to unconventional feed sources.

Blood is used for assessing the nutritional status, health and disease condition of the animals (Islam et al. 2004). Blood samples were analysed to ascertain the effect of dietary treatment on the blood profile of the animals (sheep). PCV, HB, RBC and WBC were higher in the treated groups compared to those of the control group, except in PLT, MCH, MCHC and MCV. The parameters increased as the inclusion level increased, which is clear evidence that the test ingredient has a positive impact on the health of the experimental animals and could also be attributed to the nutrients (essential nitrogen and mineral elements) available in the OPLM. The PCV values observed in this study fell within the normal range of 27%­–45% generally reported for healthy sheep (Mitruka and Rawnsley 2010; MSD, 1995). The PCV values obtained in this study were comparable to those of (26.55­–33.35) as reported by (Ogbu and Amaefule 2015) for feeding *Ocimum gratissimum* leaf meal. PCV is used as an index of the well‐being of an animal, and a reduction in the concentration of PCV, especially below the range, could suggest a lack of nutrition or the ill health of the animal (Oyawoye and Ogunkule 1998). Therefore, the obtained PCV values implied good nourishment and health of the animals.

Haemoglobin is a blood pigment that carries oxygen. Its high concentration suggests a well‐developed immune system, good carriage capacity of oxygen to various parts of the body, as well as the removal of carbon dioxide from the body of the animals (Soetan et al. 2014). The haemoglobin concentration values obtained in this study fell within the standard range of (7–15 g/dL) suggested for healthy sheep, which is an indication that the sheep have sufficient blood pigment for proper transportation of oxygen for healthy living. Also, the result implies that the use of oil palm (*Elaeis guineensis*) leaf meal in the diet of sheep had a positive effect on their health.

The RBC was significantly higher in OPLM supplemented groups, the values obtained in this present study were significantly higher than the values of 1.88­–2.31 reported by Osho et al. (2014) for Sahel goats but lower than the values of 2.86 – 3.96 as reported by Ewa et al. (2023). The values obtained for the WBC were statistically different among the treatments, as its functions are to fight infections, defend the body by phagocytosis against invasion by foreign organisms and to produce or at least transport and distribute antibodies in the immune response. Thus, animals with low WBC counts are at a higher risk of infections due to a weakened immune response. Conversely, animals with higher WBC counts, particularly those with active phagocytic cells like neutrophils and macrophages, tend to exhibit stronger innate immune responses. These cells help eliminate pathogens through phagocytosis, contributing to enhanced disease resistance and better adaptability to challenging environmental conditions (Iwuji and Herbert 2012; Kabir et al. 2011; Soetan et al. 2014).

The MCHC, MCH and MCV (%) showed significant changes among the treatment groups. The significant differences among the three parameters suggest that there were differences in corpuscular sizes, and the values obtained were similar to each treatment group. The relevance of MCHC, MCH and MCV measurement lies in their use in the diagnosis of anaemia and an index of the capacity of bone marrow to produce RBC (Allelo and Mays 1998). Agyare et al. (2014) stated that MCV, MCH and MCHC are indicators of blood level conditions; therefore, a low level is an indication of anaemia and iron deficiency. There were significant differences in platelets, and the observed differences in the mean values of platelets might have been the cause of the varied degree of blood clotting of the sheep during transportation to the laboratory. Variations in the values obtained might also be attributed to factors such as iron deficiency, chronic haemolytic anaemia, infections and chronic diseases (Baker et al. 2000).

The dressing percentage values obtained in this experiment ranged between 56.05% and 62.66%, which is very much higher than the values (43.80%–47.30%; 39.99%–45.41%; 50.02%–55.88%) reported by Ukanwoke, Ibeabuchi, and Ukachukwu (2009), Aliyu et al. (2007), and Omojola and Attah (2006) who fed cassava peel meal, OPLM and combination of the above ingredients, respectively. Changes in the values of different dressing percentages in this experiment and factors that could influence it are all attributed to the difference in breed, nutrition, final body weight, fleece and hide weight, alimentary tract size, slaughtering procedure and partitioning of body fat, dressing method as some parts which are considered offal may not be considered offal in some dressing methods, place and person/people (Fasae et al. 2007). Age, nutrition, sex, pre‐slaughter weight, gut fill and slaughter byproducts are some of the factors that affect carcass characteristics (Vergara et al. 1999). The carcass weight attained in this experiment ranged from 8407 to 9307, which implies that the sheep better utilised feed for meat production, especially experimental animals under 10% OPLM.

The weight of the head, neck, legs, thigh, heart, lungs, liver, kidneys, spleen and intestines in this experiment was all expressed as a percentage of the LW. Internal organs such as the liver would vary by enlargement if there is the presence of toxins; therefore, the test ingredient is safe for the animal's consumption. The increased heart weight attained in this experiment relates to the body weight and implies good blood circulation (Frandson 1986). It also indicates that the excretory organ (kidney) was not stressed by the inclusion of the test ingredients; thus, its excretory functions were not impaired in any way (Ngi 2012).

One of the constraints of this study was the limited sample size, which was determined based on practical considerations, including resource availability and ethical concerns. Due to the high mortality rates among ruminants in the study area at the time of the experiment, sourcing weaner sheep was particularly challenging. As a result, slaughtering three out of five sheep per group was necessary to balance statistical reliability while minimising unnecessary animal loss. To ensure representativeness, a random selection process was employed, and appropriate statistical methods were incorporated to enhance the validity of the findings. However, despite these measures, the relatively small sample size may have affected the statistical power and generalizability of the results. Future studies with larger sample sizes may provide more robust conclusions.

## 4 Conclusion

OPLM demonstrates strong potential as a feed ingredient for ruminants, particularly sheep, due to its high dry matter, appreciable crude protein and balanced crude fibre content, which enhance nutrient absorption, rumen health and growth performance. Ten percent OPLM inclusion improves body weight gain and feed efficiency, while 20% inclusion negatively impacted weight and carcass traits, suggesting an optimal limit. Dressing percentage and carcass weight were higher at 10% OPLM inclusion, highlighting its efficiency for meat production. Overall, OPLM enhances growth, health and feed efficiency in sheep, making it a viable dietary component for sustainable livestock production. While higher inclusion levels (20%) showed negative effects on weight and carcass traits, 10% OPLM supplementation demonstrated improved performance without adverse health effects, supporting its suitability for sheep nutrition.

## Author Contributions

**Paulinus Ogbonnaya Essen**: conceptualization, methodology, data curation, formal analysis, investigation, validation, software, resources, project administration, visualization, writing–original draft, writing–review and editing. **Godwin Nnamdi Njoku**: data curation, investigation, validation, visualization, resources. **Godswill Uzoma Onya**: validation, conceptualization, software, data curation, investigation. **Blessing Ngozi Zachary**: data curation, investigation, resources, validation, conceptualization. **Dondo Stephen Donkoh**: conceptualization, investigation, visualization, software, resources. **Timothy Tartenger Kuka**: conceptualization, supervision, data curation, investigation, methodology, resources, visualization, writing–review and editing. **Benjamin Adjei‐Mensah**: data curation, supervision, conceptualization, software, resources, validation, formal analysis, writing–review and editing.

## Ethics Statement

All experimental protocols were reviewed and approved by the Ethics Committee of the Federal College of Agriculture Ishiagu Nigeria, a branch of the National Ethics Committee for the control and supervision of experiments on animals.

## Conflicts of Interest

The authors declare no conflicts of interest.

## Peer Review

The peer review history for this article is available at https://www.webofscience.com/api/gateway/wos/peer‐review/10.1002/vms3.70581.

## Data Availability

All data generated and/or analysed during the current study are available from the corresponding author upon reasonable request.
